# Diabetic Myopathy and Mechanisms of Disease

**DOI:** 10.4172/2167-0501.1000e179

**Published:** 2015-10-31

**Authors:** Erick O. Hernández-Ochoa, Camilo Vanegas

**Affiliations:** Department of Biochemistry and Molecular Biology, University of Maryland School of Medicine, 108 N. Greene St., Baltimore, MD, 21201, USA

## Editorial

Humans build muscle mass over the first two decades of life; begin to lose muscle mass and strength between the third and fourth decades, and the decline accelerates during the sixth decade [1,2]. Sarcopenia and dynapenia are age-related loss of skeletal muscle mass and muscle strength, respectively [1–3]. Loss of muscle mass and strength are associated with a reduction in vitality, manifested as poor mobility and physical function [3], and are accentuated by many common chronic diseases including long-term diabetes. Diabetes mellitus (DM) is characterized by chronic hyperglycemia due to insulin deficiency, resistance, or both. Late complications affect patients’ quality of life and longevity, and changes in morbidity and mortality result in major health costs. The pathogenesis and the clinical history of both type-1 (T1D) and type-2 (T2D) diabetes drastically differ; however, the resultant syndromes and complications often overlap [4]. A common feature of both T1D and T2D is a failure to preserve muscle mass and function, and is termed diabetic myopathy [5,6]. This extremely significant, but often overlooked complication is believed to contribute to the progression of additional diabetic complications due to the key role of skeletal muscle on locomotion and glucose homeostasis [7–9]. Accelerated sarcopenia and dynapenia are typical findings in elderly people with long-term T2D. Large-scale studies of elderly people with long-term T2D have shown accelerated loss of muscle mass and strength when compared to healthy counterparts [10,11]. Despite the wealth of information related to sarcopenia and dynapenia [1–3,12–25], the specific triggering events associated with loss of skeletal muscle mass and strength in older adults with diabetes remain unknown [3]. Sarcopenia, dynapenia, and T2D increase with age, and these conditions often remain unrecognized, since ~27% of subjects with T2D are still undiagnosed (National Diabetes Fact Sheet, 2011) and sarcopenia and dynapenia currently receive little attention in the clinical setting [1,25,26]. Both sarcopenia and dynapenia have been linked to elevated healthcare costs [1,25,27]. Moreover, the absolute costs associated with diabetes, sarcopenia and dynapenia are likely to rise sharply in the coming decades considering that the total number of persons over 65 years is expected to double over the next 20 years (Federal Interagency Forum on Aging-Related Statistics, 2010).

In mammalian cells, glucose is not freely permeable across the lipid bilayer, but enters by facilitated diffusion, a process in which specific integral membrane proteins passively transport glucose down a concentration gradient [28]. Blood glucose levels are closely regulated in healthy individuals and rarely stray outside the range of 4.2–6.4 mM. However, glucose values can reach as high as 7–25 mM in individuals with diabetes and in animal models of diabetes [29–33]. Hyperglycemia can be elevated for periods between insulin injections in patients with T1D, and although less severe, is often persistent in patients with T2D. Hyperglycemia is commonly found to be even more extreme (33–66 mM) in patients with uncontrolled diabetes. During such extreme events, life-threatening acute metabolic complications of diabetes such as hyperglycemic hyperosmolar state (HHS) can occur [34,35]. HHS occasionally coincides with the breakdown of muscle fibers (rhabdomyolysis) [34–37]. HHS is typically observed in elderly patients with T2D, but is diagnosed with increasing frequency in obese children [34, 35]. Although HHS is a rare condition, the reported mortality ranges as high as 20–30%. The exact mechanism(s) that causes rhabdomyolysis in a HHS remains unclear.

Mechanisms of hyperglycemic injury vary between cell types. Several of the well-known pathologic intracellular pathways directly associated with hyperglycemia include polyol pathway flux via aldose reductase activity [38], oxidative stress [39], protein glycosylation [40], and abnormal Ca2+ signaling [41]. Glucose levels in patients with type-2 diabetes can reach abnormal high levels >120–1200 mg/dL (>7–66 mM/L), changing the osmolarity significantly. For instance, modest but significant and sustained changes in osmolarity are observed in patients with long-term moderate T2D (295–315 mOsm/kg), whereas more significant changes in osmolarity (315–360 mOsm/kg) are seen during uncontrolled T1D and T2D, when compared to healthy counterparts (285–295 mOsm/kg) [33–35]. Therefore, it is likely that adaptive and/or deleterious effects of hyperglycemic osmotic stress play a role in the pathophysiology of diabetes.

While several studies have investigated the link between changes in skeletal muscle function and mass, skeletal muscle progenitor cells, muscle growth, development, repair, and metabolic activity in different models of diabetes mellitus [1–3,12–25], few have examined the impact of hyperglycemic osmotic stress. New insights into the consequences of hyperglycemic osmotic stress in diabetes have revealed the involvement of the NFAT5, a tonicity-responsive transcription factor [42,43], as an important signaling molecule in diabetes [44]. NFAT5 is a key regulator in protection from hypertonic stress in kidney epithelial cells from the renal medulla [43,44] and other cell types [43,45–54]. It is clear that many questions remain regarding the physiological or pathophysiological impact of NFAT5 in muscle performance and function. How do skeletal muscle cells cope with the exposure to phasic, persistent or extreme extracellular glucose concentrations? What are the long-term effects of diabetes on muscle architecture and performance? Does NFAT5 enhance the manifestations of this disease? These are some of the questions to be seeded in this editorial. Further knowledge of the biochemical and molecular mechanisms involved in the onset and progression of sarcopenia and dynapenia is critical for the development of targeted pharmacological tools to ameliorate diabetic myopathy and other muscle diseases. Biochemistry and Pharmacology is an open access journal with a wide scope in biomedical sciences. New studies in the field of muscle biochemistry and signaling pathways will provide novel insights to further our understanding of skeletal muscle biology and muscle diseases.
